# Cholinergic-like neurons and cerebral spheroids bearing the PSEN1 p.Ile416Thr variant mirror Alzheimer's disease neuropathology

**DOI:** 10.1038/s41598-023-39630-4

**Published:** 2023-08-08

**Authors:** Nicolas Gomez-Sequeda, Miguel Mendivil-Perez, Marlene Jimenez-Del-Rio, Francisco Lopera, Carlos Velez-Pardo

**Affiliations:** Grupo de Neurociencias de Antioquia, Instituto de Investigaciones Médicas, Facultad de Medicina, Universidad de Antioquia (UdeA), Calle 70 No. 52-21, and Calle 62 # 52-59, Torre 1, Laboratorio 412, Medellín, Colombia

**Keywords:** Cell biology, Neuroscience

## Abstract

Familial Alzheimer’s disease (FAD) is a complex neurodegenerative disorder for which there are no therapeutics to date. Several mutations in presenilin 1 (PSEN 1), which is the catalytic component of γ-secretase complex, are causal of FAD. Recently, the p.Ile416Thr (I416T) PSEN 1 mutation has been reported in large kindred in Colombia. However, cell and molecular information from I416T mutation is scarce. Here, we demonstrate that menstrual stromal cells (MenSCs)-derived planar (2D) PSEN 1 I416T cholinergic-like cells (ChLNS) and (3D) cerebral spheroids (CSs) reproduce the typical neuropathological markers of FAD in 4 post-transdifferentiating or 11 days of transdifferentiating, respectively. The models produce intracellular aggregation of APPβ fragments (at day 4 and 11) and phosphorylated protein TAU at residue Ser^202^/Thr^205^ (at day 11) suggesting that iAPPβ fragments precede p-TAU. Mutant ChLNs and CSs displayed DJ-1 Cys^106^-SO_3_ (*sulfonic acid*), failure of mitochondria membrane potential (ΔΨ_m_), and activation of transcription factor c-JUN and p53, expression of pro-apoptotic protein PUMA, and activation of executer protein caspase 3 (CASP3), all markers of cell death by apoptosis. Moreover, we found that both mutant ChLNs and CSs produced high amounts of extracellular eAβ_42_. The I416T ChLNs and CSs were irresponsive to acetylcholine induced Ca^2+^ influx compared to WT. The I416T PSEN 1 mutation might work as dominant-negative PSEN1 mutation. These findings might help to understanding the recurring failures of clinical trials of anti-eAβ_42_, and support the view that FAD is triggered by the accumulation of other intracellular AβPP metabolites, rather than eAβ42.

## Introduction

Presenilin 1 (PSEN1) is an intramembrane aspartyl protease^1^ which is the core of the γ-secretase complex responsible for amyloid-β (Aβ) generation^2,3^. Five PSEN1 mutations (e.g., p.Met146Leu, p.His163Arg, p.Ala246Glu, p.Leu286val, p.Cys410Tyr) were originally discovered in 1995 on human chromosome 14 (14q24.3) by genetic analysis of six large pedigrees with Alzheimer disease (AD)^4^. Since then, more than 300 mutations in PSEN1 have been reported (https://www.alzforum.org/mutations/psen-1) and are associated with the most common cause of early-onset familial Alzheimer's disease (FAD)^5^. Among those variants, the missense p.Glu280Ala (p.E280A) mutation was discovered in large Colombian kindred^6^. The PSEN1 p.E280A mutation of European ancestry^7^, also known as the paisa mutation^8^, affects nearly 1,000 individuals with about 400 confirmed carriers. Since the Colombian kindred has shown devoted participation and adherence in longitudinal studies, the effects of this mutation have been investigated extensively in connection not only with genetic^9,10^, cellular^11–13^, neuropathologic^14,15^, neuropsychologic^16,17^, and neurologic analyses^18–21^ but also in biomarker progression^22–25^ and prevention clinical trials (ClinicalTrials.gov Identifier: NCT01998841;^26^). Interestingly, the classical pathogenesis of the PSEN1 E280A mutation have been modeled in fibroblast-derived human induced pluripotent stem cells (hiPSCs)^11^, in Warthon Jelly MSCs-derived cholinergic-like neuronal cells (ChLNs)^27^, and in menstrual MSCs-derived cerebral spheroids (CSs)^28^. Specifically, the PSEN1 p.E280A ChLN cells and CSs displayed an increased secretion of extracellular Aβ, mostly eAβ_42_ peptide fragment, intracellular aggregation of soluble amyloid precursor protein beta fragments (iAPPβf), oxidative stress (OS, i.e., oxidation of the stress sensor protein DJ-1 Cys^106^SO^−^ into Cys^106^SO_3_), loss of mitochondrial membrane potential (ΔΨ_m_), hyperphosphorylation of the protein tau (at residue Ser^202^/ Thr^205^), dysregulation of intracellular Ca^2+^ influx, and apoptosis^27,28^. Although the exact mechanism by which PSEN1 (/γ-secretase) mutations generate high amounts of the extracellular amyloidogenic Aβ_42_ fragment is not yet well-established^29–31^, the PSEN1 p.E280A works as dominant-negative PSEN1 mutation^32,33^, thereby triggering intracellular and extracellular Aβ-dependent signaling leading to neuronal death.

Unfortunately, in addition to the PSEN1 p.E280A mutation, 10 other PSEN1 mutations of European (five), Native American (three), undetermined (1), and African (one) ancestry have been reported in Colombia^34^. Specifically, a novel missense PSEN1 Ile416Thr (Chr14:73683951T > C; c.1247T > C; p.I416T) pathogenic variant of African ancestry that causes AD in large kindred has been reported in the Antioquia region^35^ (for a journalistic version see^36^). Remarkably, the cerebral pattern of Aβ deposition and the PET-tau scans in I416T carriers resembled those previously reported in PSEN1 p.E280A carriers^37,38^. Likewise, neuropsychological evaluation of symptomatic carriers presented a mean age at onset of 42.35 ± 6.28 years for memory complaints, 47.6 ± 5.83 years for mild cognitive impairment (MCI), and the mean age of onset of dementia was 51.6 ± 5.03 years as well as depression, anxiety, delusions, hallucinations, and insomnia analysis also resembled those reported for the E280A mutation^8,39^. These observations suggest that both I416T and E280A are genetically and phenotypically homologous variants independently of their ancestry origin. However, whether the I416T variant expresses cellular and/ or molecular markers similarly to the E280A is still unknown. Answering this question is not a minor issue because this information might serve to answer whether potential treatment developments and/ or prevention clinical trials discovered in E280A might also apply to I416T patients. Furthermore, depending on the Aβ profiles generated by the different PSEN1 variants, it is possible to determine the pathogenicity of a particular mutation and predict age at disease onset^40^. Based on the above considerations, and the fact that both mutations are 100% penetrant^8,35^, we posit that the I416T behaves molecularly like the E280A paisa mutation in cell models of FAD.

Endometrial mesenchymal stem cells (enMSCs) are a class of adult stem cells with self-renewal capacity, differentiation potential, low immunogenicity, low tumorigenicity, among other biological characteristics^41,42^. Specific surface markers (e.g., CD140b/ CD146 co-expression, SUSD2) have shown their perivascular identity in the endometrium, including the layer which sheds during menstruation. Cells with MSC properties have been identified in menstrual fluid and commonly termed menstrual blood stem/stromal cells (MenSC)^43^**.** Recently, we have validated MenSCs as a reliable source of planar (2D) ChLNs and cerebral spheroids (3D CSs) or neurospheres^44^. Since the protocol to obtain mutant hiPSCs take no less than 35 days^45,46^, we selected PSEN1 I416T MenSCs as an experimental biological source, which presents minimal technical limitations, and minor ethical issues. Moreover, MenSCs-derived ChLNs and CSs display the AD neuropathological hallmarks by day 11^27,28^. In the present work, we aimed to establish an in vitro MenSCs-derived neuronal-like model bearing the mutation I416T. Like the E280A, we found that PSEN1 I416T mutation negatively affects the structure and functionality of MenSCs-derived planar (2D) ChLNs and 3D CSs expressing high iAPPβf aggregates and high amounts of eAβ_42_, and hyperphosphorylated protein tau. Moreover, PSEN1 I416T also showed loss of ΔΨ_m_, high production of reactive oxygen species (ROS, i.e., H_2_O_2_), signs of apoptosis death, and alterations in Ca^2+^ influx (Table 1). Therefore, we anticipate that experimental treatment approaches for FAD E280A^47^ might be also valid for FAD I416T.

**Table 1:** Comparison of the effects of mutation E280A and I416T on cholinergic-like neural cells (2D) and on (3D) cerebral spheroids from human umbilical cord mesenchymal stromal cells (UC-MSCs) and human menstrual stromal cells (MenSCs).

Condition > effect	Intracelular						Extracelular	Functionality
	iAPPβf	oxDJ-1 Cys^106^	ΔΨ_m_		CASP-3	p-TAU (Ser^202^/ Thr^205^)	eAβ_42/_eAβ40	[(Ca^2+^)_i_]
E280A (2D)^#^	(↑)	(↑)	(↓)	(↑)		(↑)	(↑)	(↓)
E280A (3D)^$^	(↑)	(↑)	(↓)	(↑)		(↑)	(↑)	(↓)
I416T (2D)*	(↑)	(↑)	(↓)	(↑)		(↑)	(↑)	(↓)
I416T (3D)**	(↑)	(↑)	(↓)	(↑)		(↑)	(↑)	(↓)
WT*,**	(−)	(−)	(−)	(−)		(−)	(−)	(↑)

(↑) = increases; (↓) = reduces; ( −) = no effect.

^#^Ref.^27^.

^$^Ref.^28^.

*This work.

**This work.

## Results

### Wild-type and PSEN1 I416T MenSCs differentiated into mesodermal and transdifferentiated into ectoderm lineages

**Figure 1:**
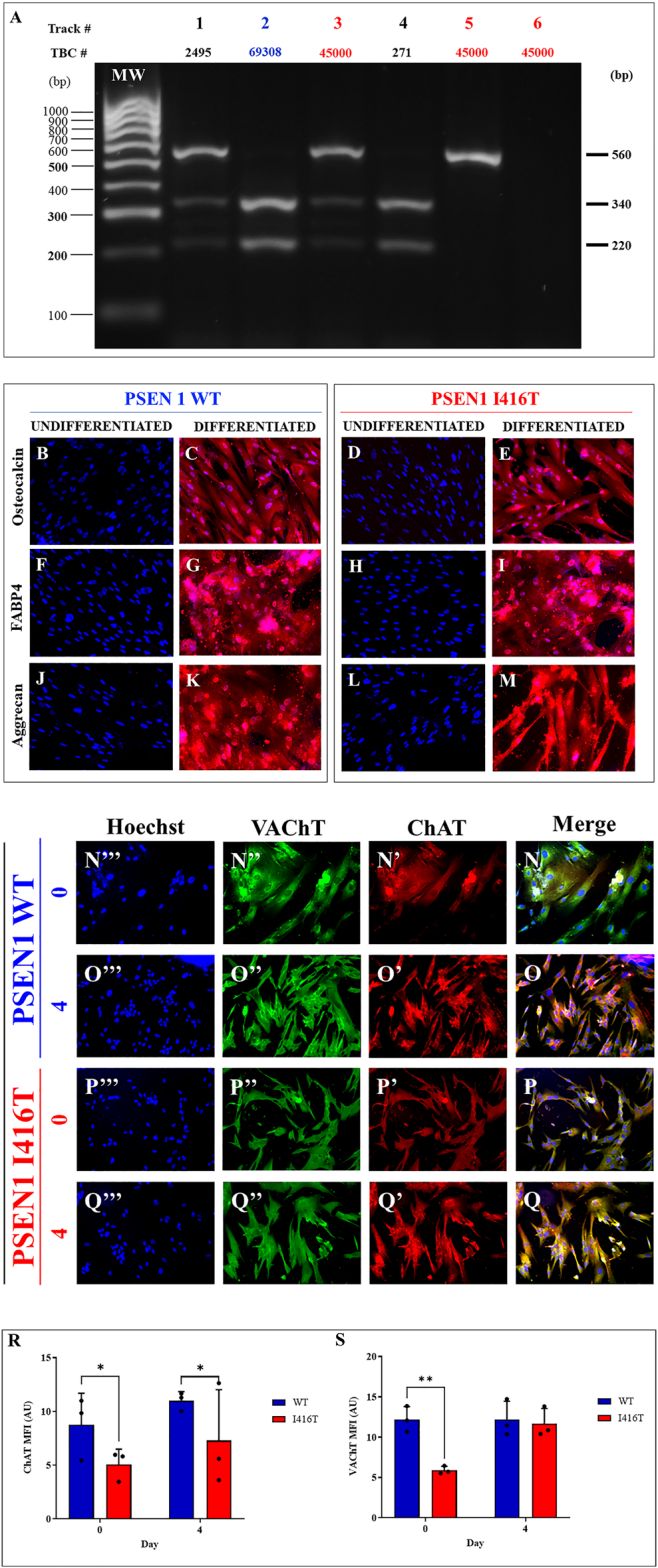
(**A**) Agarose gel electrophoresis for the I416T mutation isolated from samples of mutation carriers. PCR products from blood samples were subjected to restriction endonuclease digestion analysis using AseI enzyme, resolved on 2% agarose gel electrophoresis, and visualized with red gel under ultraviolet illumination. The size of the amplicon was 560 bp. The 340 and 220 bp fragments correspond to the wild-type phenotype, and the 560, 340, and 220 fragments are for the mutant heterozygous phenotype. (M) Molecular weight marker; track (1) positive control with the I416T mutation (Code 2495); track (2) Wild phenotype negative control (Code 69,308); track (3) mutation carrier (Code 45,000); track (4) mutation carrier E280A (Code 271); track (5) control of digestion (Code 4500, without the AseI enzyme); track (6) PCR control (without template). The original gel is presented in Supplementary Fig. 1. (**B**, **D**) Osteocalcin negatively stained undifferentiated MenSCs grown on regular culture medium. (**C**, **E**) Osteocalcin positively stained osteoblasts differentiated from MenSCs. (**F**, **H**) FABP4 negatively stained undifferentiated MenSCs grown on regular culture medium. (**G**, **I**) FABP4 positively stained adipocytes differentiated from MenSCs. (**J**, **L**) Aggrecan negatively stained undifferentiated MenSCs grown on regular culture medium. (**K**, **M**) Aggrecan positively stained chondrocytes differentiated from MenSCs. Image magnification, 20x. The images represent 1 out of 3 independent experiments. (**N**–**Q**) MenSCs transdifferentiated into cholinergic-like neurons. WT PSEN1 and PSEN1 I416T MenSCs were cultured in a cholinergic differentiation medium as described in the Materials and Methods section for 7 days. Thereafter, WT PSEN1 and PSEN1 I416T ChLNs were left in regular culture medium (RCm) for 0 and 4 days. Then cells were double-stained with primary antibodies against ChAT (red fluorescence; **N’**–**Q’**) and VAChT (green fluorescence; **N’’**–**Q’’**). The nuclei are stained with Hoechst 33,342 (blue fluorescence; **N’’’**–**Q’’’**). (**R**) Quantification of ChAT fluorescence intensity. (**S**) Quantification of VAChT fluorescence intensity. The figures represent 1 out of 3 independent experiments. One-way ANOVA, post hoc test Šidák. Data are presented as mean ± SD (*p < 0.05; **p < 0.01; ***p < 0.001).

We first verified the genetic presenilin gene status of the MenSCs' donators. As shown in Fig. 1A, restriction fragment length polymorphism analysis (RFLP- VspI (AseI)) of a positive (control) heterozygous for the mutation PSEN1 I416T reveals three fragments of 560, 340, and 220 bp (track #1), whereas WT PSEN1 produces two fragments of 340, and 220 bp only (track #2). The change of a T>C in codon 416 (i.e., ATT to ACT) generates a second cutting in the exon 11 of PSEN1 recognized by the restriction enzyme VspI (AseI). Based on this observation, we confirmed that the MenSCs sample (TBC# 45000) presents the PSEN1 I416T mutation (track #3). We also confirm that the DNA sample from a mutation carrier E280A (TBC#271) was negative for the PSEN1 I416T (track# 4). As a quality control, we incubated an aliquot of DNA with the PSEN1 I416T mutation (TBC# 45000) without RE VspI (AseI) (track # 5, 540 bp band), or without DNA templates (or primers, Track# 6, no bands).

Since the MenSCs can differentiate into mesodermal and ectoderm cell lineages, we incubated WT and PSEN1 I416T MenSCs with their respective adipogenic, osteogenic, chondrogenic, and cholinergic medium according to standard protocols^44^. As expected, no differentiation markers were displayed in both WT and mutant MSCs when cultured in regular culture medium (RCm, Fig. 1B,D,F,H,J,L), whereas WT and mutant MenSCs differentiated into either osteocyte (Fig. 1C,E), adipocyte (Fig. 1G,I), or chondrocyte cells (Fig. 1K,M) according to osteocalcin, fatty acid binding protein 4 (FABP4), and aggrecan positive cells, respectively. Furthermore, MenSCs were also able to transdifferentiate into cholinergic-like neuronal cells (ChLNs, Fig. 1N–Q) according to cholinergic marker ChAT (Fig. 1R) and VAChT (Fig. 1S).

### PSEN1 I416T ChLNs but not PSEN1 WT accumulates sAPPβf, induces loss of ΔΨ_m_, shows oxidized oxidative sensor protein DJ-1, and high generation of reactive oxygen species (ROS)

**Figure 2:**
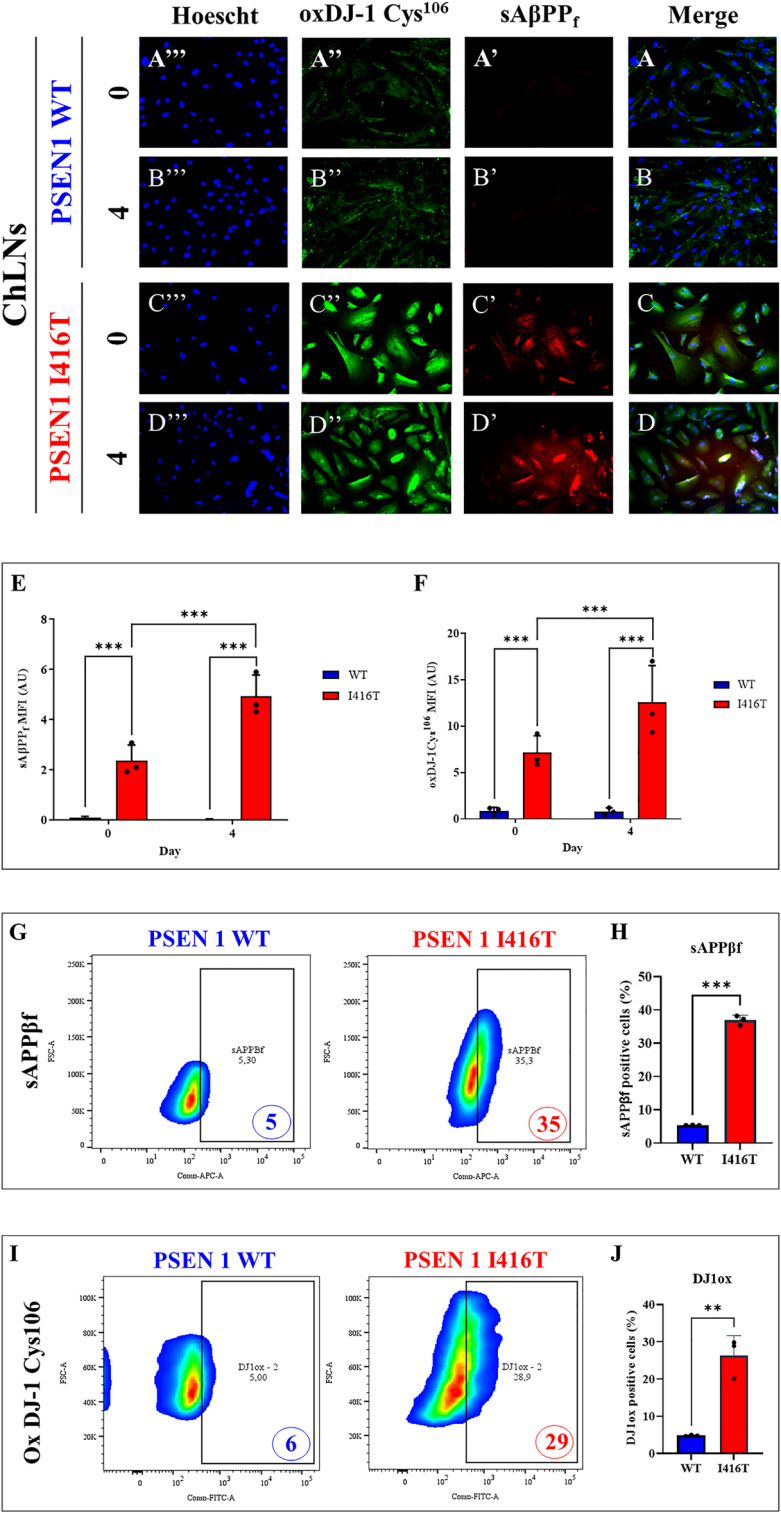
PSEN1 I416T Cholinergic-Like Neurons (ChLNs) show high levels of intracellular sAPPβf and oxidized DJ-1. WT PSEN1 and PSEN1 I416T MenSCs were cultured in a cholinergic differentiation medium as described in the *Materials and Methods* section for 7 days. Thereafter, WT PSEN1 and PSEN1 I416T ChLNs were left in regular culture medium (RCm) for 0 and 4 days. (**A**–**D**) Cells were double stained with primary antibodies against APP751/Aβ42 (red fluorescence; **A’**–**D’**) and oxDJ-1Cys^106^ (green fluorescence; **A”**–**D”**). The nuclei were stained with Hoechst 33,342 (blue fluorescence; **A”’**–**D”’**). (**E**) Quantification of sAPPβf by fluorescence intensity. (**F**) Quantification of oxDJ-1Cys106 fluorescence intensity (n = 3). (**G**) Quantification of sAPPβf by flow cytometry. (**H**) Quantification of sAPPβf by flow cytometry (n = 3). (**I**) Quantification of oxDJ-1 by flow cytometry. (**J**) Quantification of oxDJ-1 by flow cytometry (n = 3). Data are expressed as the mean ± SD; *p < 0.05; **p < 0.01; ***p < 0.001. The histograms and figures represent 1 out of 3 independent experiments. Image magnification, 200x.

**Figure 3:**
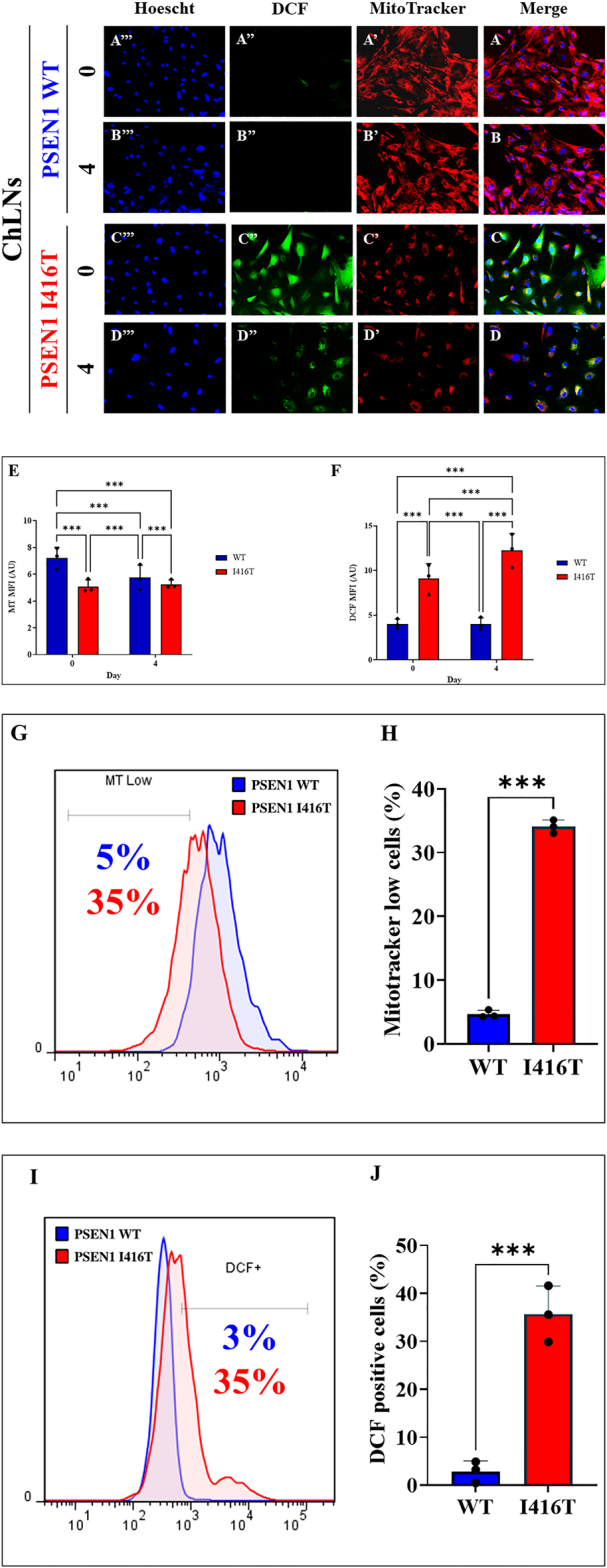
PSEN1 I416T Cholinergic-Like Neurons (ChLNs) show high mitochondrial membrane potential (ΔΨm), and high levels of intracellular reactive oxygen species (ROS). WT PSEN1 and PSEN1 I416T MenSCs were cultured in a cholinergic differentiation medium as described in the Experimental Procedure section for 7 days. Thereafter, WT PSEN1 and PSEN1 I416T ChLNs were left in regular culture medium (RCm) for 0 and 4 days. (**A**–**D**) Cells were double stained with MitoTracker™ Red FM (red fluorescence; **A’**–**D’**) and DCF (green fluorescence; **A”**–**D”**). The nuclei were stained with Hoechst 33,342 (blue fluorescence; **A”’**–**D”’**). (**E**) Quantification of ΔΨm by fluorescence intensity. (**F**) Quantification of DCF fluorescence intensity (n = 3). (**G**) Representative histogram showing ΔΨm by flow cytometry. (**H**) Quantification of ΔΨm by flow cytometry (n = 3). (**I**) Representative histogram showing DCF + by flow cytometry. (**J**) Quantification of DCF by flow cytometry (n = 3). Data are expressed as the mean ± SD; *p < 0.05; **p < 0.01; ***p < 0.001. The histograms and figures represent 1 out of 3 independent experiments. Image magnification, 200x.

**Figure 4:**
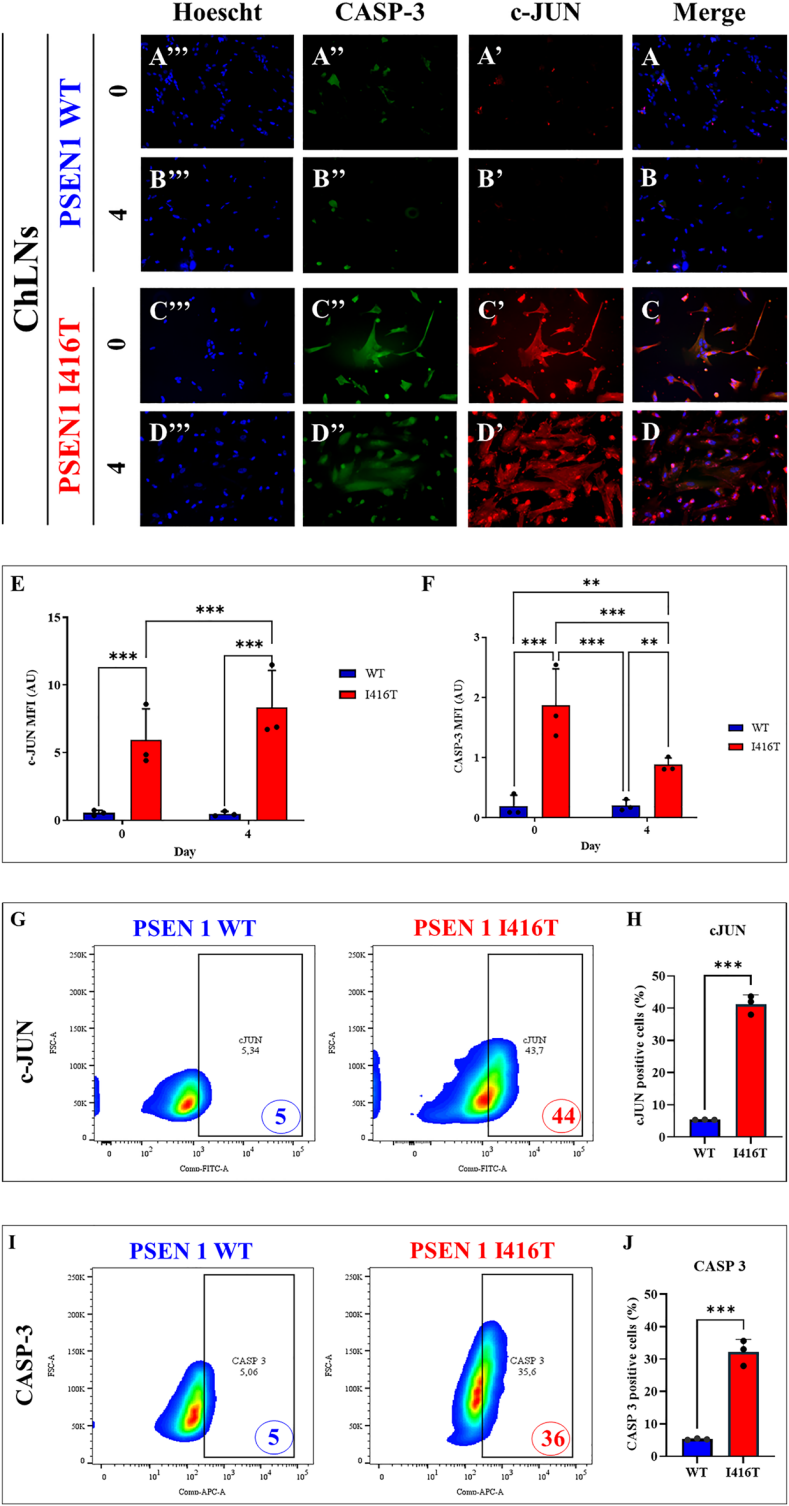
PSEN1 I416T ChLNs display activation c-JUN and CASPASE-3. WT PSEN1 and PSEN1 I416T MenSCs were cultured in a cholinergic differentiation medium as described in the *Materials and Methods* section for 7 days. Thereafter, WT PSEN1 and PSEN1 I416T ChLNs were left in regular culture medium (RCm) for 0 and 4 days. (**A–D**) Cells were double stained with c-JUN (red fluorescence; **A’**–**D’**) and CASPASE-3 (green fluorescence; **A”**–**D”**). The nuclei were stained with Hoechst 33,342 (blue fluorescence; **A”’**–**D”’**). (**E**) Quantification of c-JUN by fluorescence intensity. (**F**) Quantification of CASPASE-3 fluorescence intensity (n = 3). (**G**) Quantification of c-JUN by flow cytometry. (**H)** Quantification of c-JUN by flow cytometry (n = 3). (**I**) Quantification of CASPASE-3 by flow cytometry. (**J**) Quantification of CASPASE-3 by flow cytometry (n = 3). Data are expressed as the mean ± SD; *p < 0.05; **p < 0.01; ***p < 0.001. The histograms and figures represent 1 out of 3 independent experiments. Image magnification, 200x.

Next, we wanted to determine whether mutant neuronal-like cells express neuropathologic markers of AD. To this aim, we cultured ChLN cells for 0- and 4-days post-transdifferentiating in RCm. Then amyloid, mitochondria, OS markers were evaluated. As shown in Fig. 2, neither iAPPβf (Fig. 2A',B') nor oxidized DJ-1 protein (Fig. 2A'',B'') was detected in WT ChLNs at day 0 (Fig. 2A,E) and 4 (Fig. 2B,F), respectively. In contrast, I416T ChLNs show sAPPβf aggregates (Fig. 2C', D',E) and oxDJ-1 (Fig. 2C'',D'',F) at day 0 and 4 post-transdifferentiating (Fig. 2E,F). Indeed, the amount of iAPPβf and oxDJ-1 almost doubles at day 4 post-transdifferentiating. Flow cytometry analysis on day 4 (Fig. 2G–J) showed that the amount of sAPPβf and oxDJ-1 increased by +600% (Fig. 2G,H) and +384% (Fig. 2I,J), respectively compared to WT ChLNs. When the generation of ROS and ΔΨ_m_ was evaluated in those cells, we found high ΔΨ_m_ (Fig. 3A',B') but no ROS (Fig. 3A'',B'') in WT ChLNs by day 0 and 4 (Fig. 3A,B,E,F) according to immunocytochemistry analysis. The mutant ChLNs (Fig. 3C,D) show a significant reduction in ΔΨ_m_ at day 0 (1.41-fold decrease, Fig. 3C’,E) and at day 4 (1.10-f d, Fig. 3D’,E), but a significant increase in ROS generation (2.25-fold increase and 3.0-f i, Fig. 3C'',D'',F). A similar trend of observations was obtained by flow cytometry on day 4 (Fig. 3G–J). Indeed, the ΔΨm in mutant cells diminished -86% (Fig. 3G,H), whereas ROS production increased +1067% compared to WT PSEN 1 (Fig. 3I,J).

### PSEN1 I416T ChLNs express activated c-JUN and CASPASE-3

It is known that ROS/ H_2_O_2_ activate c-JUN, P53, pro-apoptotic PUMA, and CASPASE-3^48^. Unsurprisingly, immunocytochemistry analysis showed that WT ChLNs display neither activated c-JUN (Fig. 4A',B'), nor CASP-3 (Fig. 4A'',B'') at day 0 (Fig. 4A,E,F) and 4 (Fig. 4B,E,F). On the contrary, we detected activated c-JUN (Fig. 4C' and 4D'), and CASP3 (Fig. 4C'',D'') at day 0 (11.0-f i: 4E, and 9.36-f i: 4F, respectively) and at day 4 (Fig. 4D, 17.42-f i: 4E, 4.20-f i: 4F, respectively) in I416T ChLNs. Flow cytometry evaluation revealed similar tendency of data at day 4 (Fig. 4G–J). Notably, c-JUN (Fig. 4G,H), and CASP3 (Fig. 4I,J), increased their expression by +780%, and +620%, respectively in PSEN 1 I416T ChLNs when compared to WT ChLNs.

### PSEN1 I416T ChLNs express activated PUMA and P53

**Figure 5:**
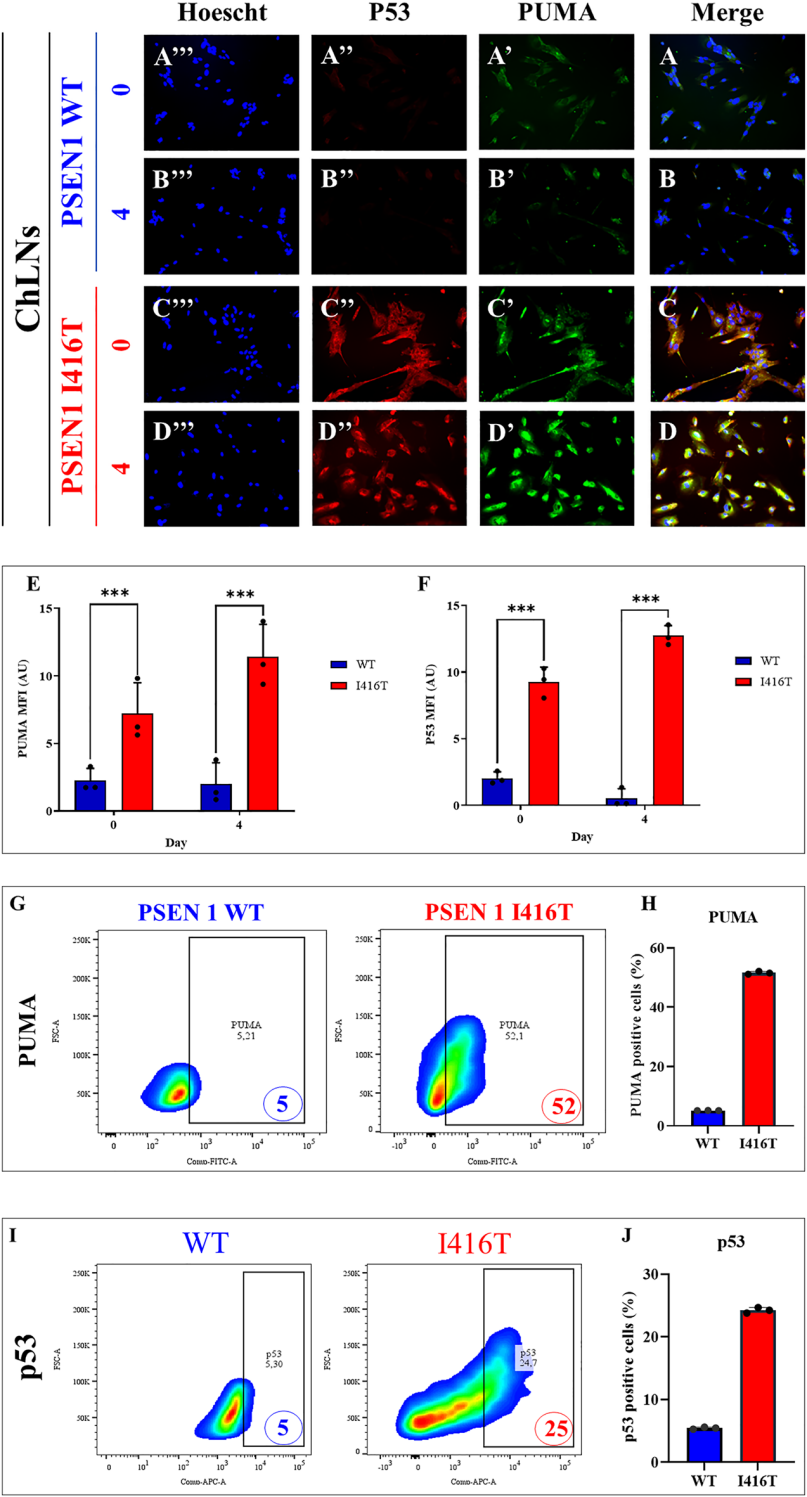
PSEN1 I416T ChLNs display activation P53 and PUMA. WT PSEN1 and PSEN1 I416T MenSCs were cultured in a cholinergic differentiation medium as described in the *Materials and Methods* section for 7 days. Thereafter, WT PSEN1 and PSEN1 I416T ChLNs were left in regular culture medium (RCm) for 0 and 4 days. (**A**–**D**) Cells were double stained with PUMA (green fluorescence; **A’**–**D’**) and P53 (red fluorescence; **A”**–**D”**). The nuclei were stained with Hoechst 33,342 (blue fluorescence; **A”’**–**D”’**). (**E**) Quantification of PUMA by fluorescence intensity. (**F**) Quantification of P53 fluorescence intensity (n = 3). (**G**) Quantification of PUMA by flow cytometry. (**H)** Quantification of PUMA by flow cytometry (n = 3). (**I**) Quantification of P53 by flow cytometry. (**J**) Quantification of P53 by flow cytometry (n = 3). Data are expressed as the mean ± SD; *p < 0.05; **p < 0.01; ***p < 0.001. The histograms and figures represent 1 out of 3 independent experiments. Image magnification, 200x.

Like c-JUN/ CASP3, we found that WT ChLNs display neither activated PUMA (Fig. 5A',B') nor P53 (Fig. 5A'',B'') at day 0 (Fig. 5A,E,F) and 4 (Fig. 5B,E,F) according to immunocytochemistry analysis. We detected activated PUMA (Fig. 5C',D'), p53 (Fig. 5C'',D'') at day 0 (Fig. 5C, 3.20-f i: E, and 4.50-f i: F, respectively), and at day 4 (Fig. 5D, 5.70-f i: E, and 23.21-f i: F, respectively) in I416T ChLNs. Notably, PUMA (Fig. 5G,H), and P53 (Fig. 5I,J) increased their expression by +940%, and +400%, respectively revealed by flow cytometry assessment at day 4 (Fig. 5G–J) in mutant ChLNs.

### PSEN1 I416T ChLNs display hyperphosphorylated protein TAU (p-TAU)

**Figure 6:**
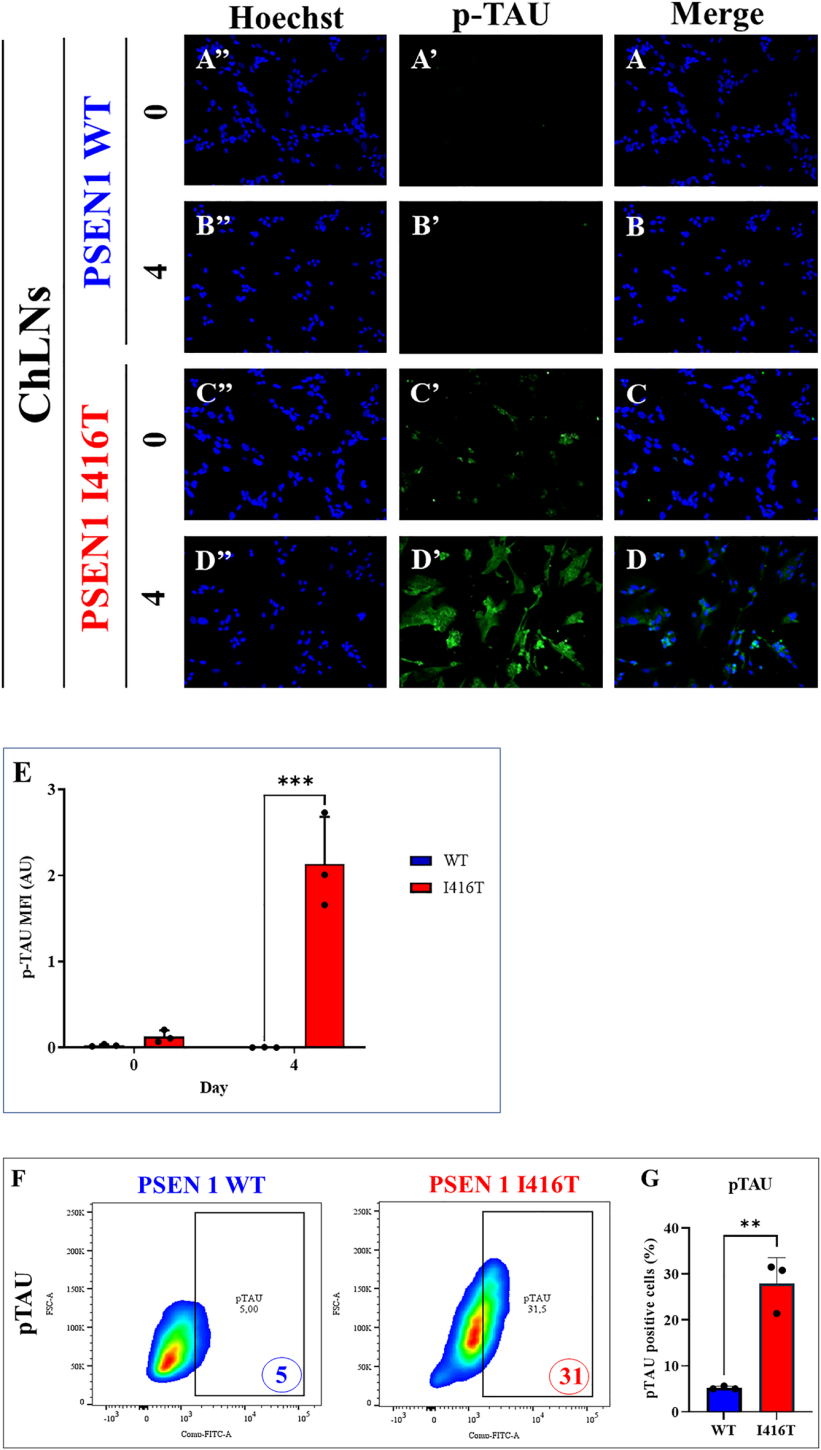
PSEN1 I416T ChLNs show high levels of phosphorylation of protein TAU. WT PSEN1 and PSEN1 I416T MenSCs were cultured in a cholinergic differentiation medium as described in the *Materials and Methods* section for 7 days. Thereafter, WT PSEN1 and PSEN1 I416T ChLNs were left in regular culture medium (RCm) for 0 and 4 days. (**A**–**D**) Cells were stained with p-TAU (green fluorescence; **A’**–**D’**). The nuclei were stained with Hoechst 33,342 (blue fluorescence; **A”**–**D”**). (**E**) Quantification of p-TAU by fluorescence intensity. (**F**) Quantification of p-TAU by flow cytometry. (**G**) Quantification of p-TAU by flow cytometry (n = 3). Data are expressed as the mean ± SD; *p  < 0.05; **p < 0.01; ***p < 0.001. The histograms and figures represent 1 out of 3 independent experiments. Image magnification, 200x.

Intracellularly hyperphosphorylated protein tau (p-TAU Ser202/Thr205) is a classical pathological marker in AD (Neddens et al., 2020). Therefore, we evaluated whether mutant ChLNs present p-TAU. Fig. 6 shows that WT ChLNs displayed no p-TAU at day 0 (Fig. 6A,E) and 4 (Fig. 6B,E). Likewise, no p-TAU was almost undetected in I416T ChLNs at day 0 (Fig. 6C,E), but the phosphorylated protein was identified in mutant ChLNs at day 4 (76.11-f i, Fig. 6D,E). Of note, p-TAU was increased by +520% in I416T cells compared to WT ChLNs according to flow cytometry analysis (Fig. 6F,G).

### PSEN1 I416T ChLNs secrete high amounts of eAβ_42_

**Figure 7:**
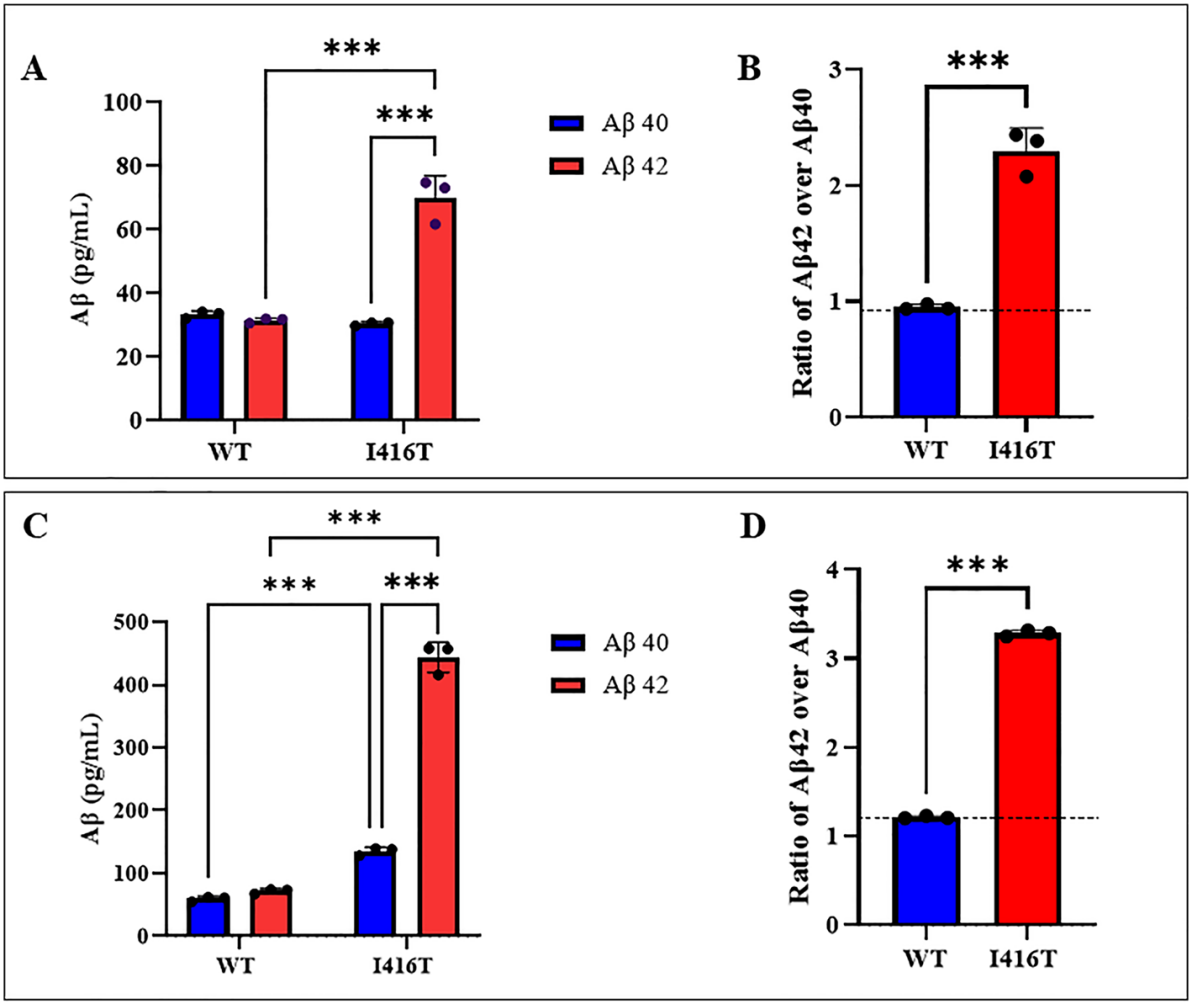
ELISA quantification of extracellular Aβ_40_ and Aβ_42_ peptide in supernatants from WT PSEN1 and I416T ChLNs and cerebral spheroids (CSs). WT PSEN1 and PSEN1 I416T MenSCs were cultured in a *cholinergic-N-Run* differentiation medium as described in the *Materials and Methods* section for 7 days. Thereafter, WT PSEN1 and PSEN1 I416T ChLNs were left in regular culture medium (RCm) for 4 days post-transdifferentiating. The levels of secreted Aβ1–42 and Aβ1–42 peptides were determined as described in *Materials and Methods* section. (**A**) ELISA measurements of supernatant from PSEN1 I416T ChLNs cells at day 4 post-transdifferentiating. (**B**) Aβ42 over Aβ40 ratio in PSEN1 I416T ChLNs compared with PSEN1 WT at day 4. WT PSEN1 and PSEN1 I416T MenSCs were cultured in a *Fast-N-Spheres* medium as described in the *Materials and Methods* section for 11 days. (**C**) ELISA measurements of supernatant from PSEN1 I416T CSs cells at day 11. (**D**) Aβ42 over Aβ40 ratio in PSEN1 I416T CSs compared with PSEN1 WT at day 11. The figures represent 1 out of 3 independent experiments. One-way ANOVA, post hoc test Bonferroni. Data are presented as mean ± SD (* *p* < 0.05; **p < 0.01; ***p < 0.001).

The molar ratio of Aβ_42_ over Aβ_40_ is extensively used as a proxy indicator for the pathogenic effect of presenilin mutations. Fig. 7A shows that WT PSEN 1 ChLNs produced a basal level of Aβ_40_ and Aβ_42,_ whereas PSEN 1 I416T ChLNs released a basal level of Aβ_40_ but high amount of Aβ_42_ (i.e., twice the amount of Aβ_40_) at 4 days of post-transdifferentiating. Therefore, mutant PSEN 1 significantly increased the amount of Aβ_42_ peptide (by +125%) compared to WT PSEN 1, resulting in a higher ratio of Aβ_42_ (2.42-fold increase, Fig. 7B).

### PSEN1 I416T ChLNs respond to ACh stimuli

**Figure 8:**
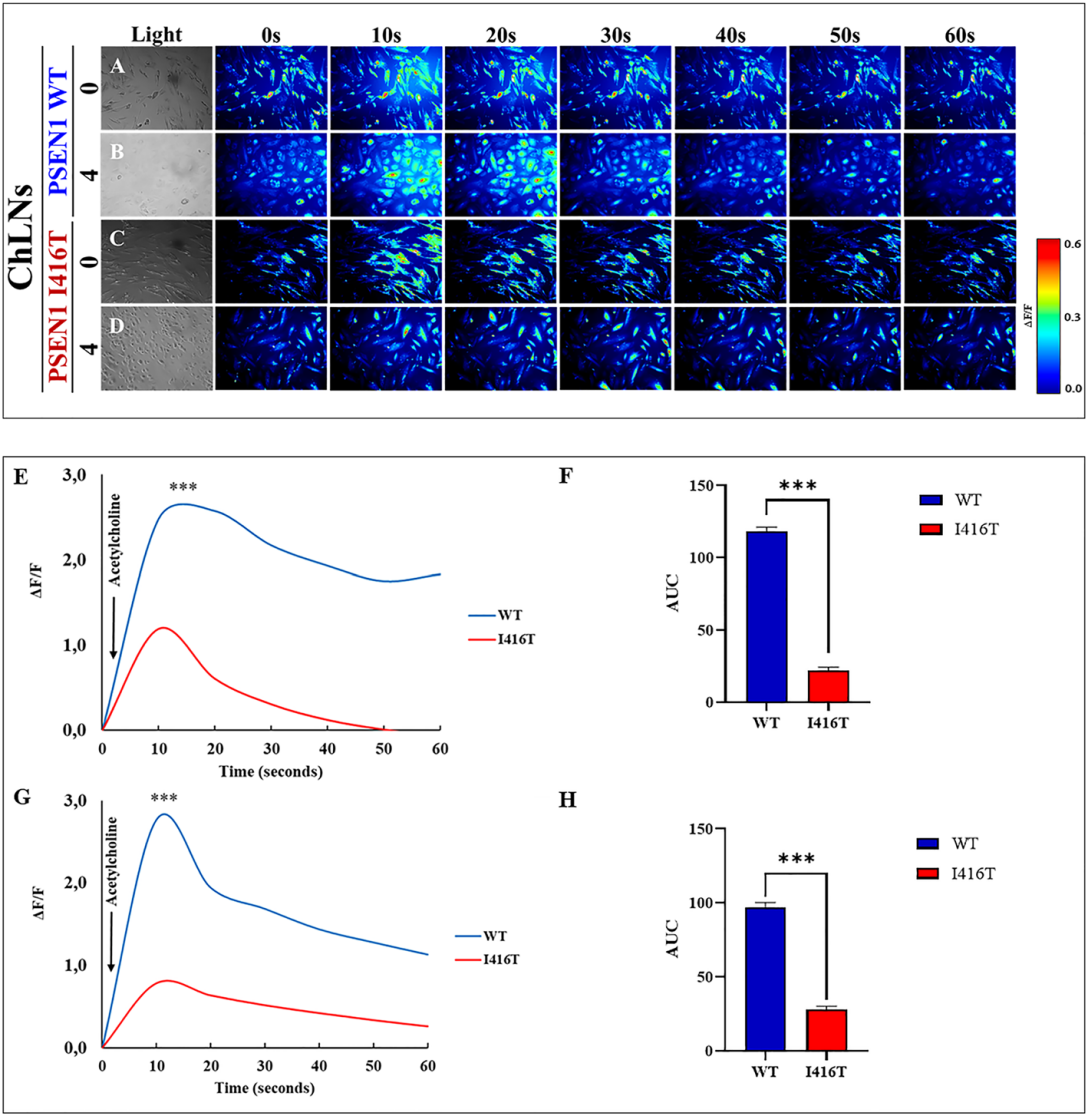
PSEN1 I416T ChLNs show a reduced functional response to Acetylcholine (ACh). After 7 days of transdifferentiating, WT PSEN1 and PSEN1 I416T ChLNs were left in regular culture medium for 0 and 4 days, as indicated in the figure. Time-lapse images (0, 10, 20, 30, 40, 60 s) of Ca^2+^ fluorescence in WT PSEN1 and PSEN1 I416T ChLNs after 0 (**A**, **C**), and 4 days (**B**, **D**) in response to ACh treatment. ACh was puffed into the culture at 0s (arrow). Then, the Ca^2+^ fluorescence of the cells was monitored at the indicated times. Color contrast indicates fluorescence intensity: dark blue < light blue < green < yellow < red. (**E**, **F**) Graph showing ΔF/F and area under the curve (AUC) of naïve and mutant cells in response to ACh treatment after 0 days. (**G**, **H**) Graph showing ΔF/F and area under the curve (AUC) of naïve and mutant cells in response to ACh treatment after 4 days post-transdifferentiating. The figures represent 1 out of 3 independent experiments. Data are expressed as the mean ± SD; *p < 0.05; **p < 0.01; ***p < 0.001.

A typical functional feature of neurons is that they respond to ACh-induced Ca^2+^ influx. We, therefore, investigated whether PSEN1 I416T ChLNs respond to ACh stimuli. To this aim, both WT and mutant PSEN1 ChLN cultures were exposed to ACh (1 mM final concentration). Fig. 8 shows that ACh induced an elevation of intracellular Ca^2+^ in WT PSEN1 ChLNs at day 0 (Fig. 8A,E, average fluorescence change (ΔF/F) = 2.58 ± 0.22), and day 4 post-transdifferentiating (Fig. 8B,G, (ΔF/F)=2.77 ± 0.14), with a mean duration of 10 s each (n = 20 ChLN cells imaged, N = 3 dishes) according to cytoplasmic Ca^2+^ responses to Fluo-3-mediated imaging (Fig. 8F,H). However, PSEN1 I416T ChLNs posed a reduced intracellular Ca^2+^ influx response to ACh treatment at day 0 (Fig. 8C,E, (ΔF/ F) =1.19 ± 0.12) and 4 (Fig. 8D,G, (ΔF/ F) = 0.78 ± 0.25, mean duration of 10 s (n = 20 ChLN cells imaged, N = 3 dishes)) compared to WT PSEN1 ChLNs (Fig. 8F,H).

### PSEN1 I416T MenSCs generate cerebral spheroids (CSs) displaying classical neuropathologic features of FAD

**Figure 9:**
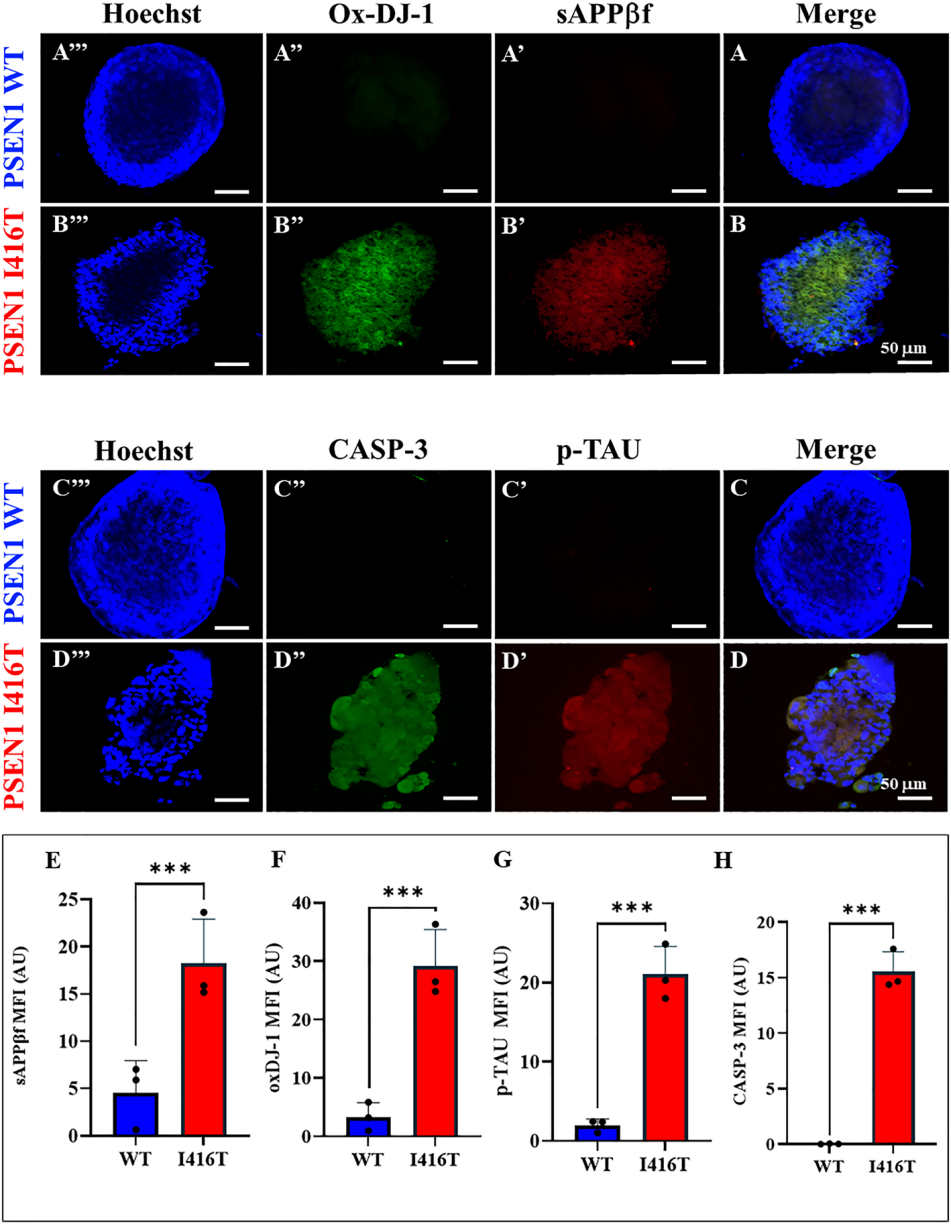
PSEN1 I416T Cerebral spheroids (CSs) show high levels of (i)sAPPβf, Ox-DJ-1, Tau phosphorylation (p-TAU) and caspase 3 (CASP3) activation. Both PSEN1 WT and I416T CSs were left in cultured for 11 days. Then, nuclei were stained with Hoechst (blue; A’’’-D’’’) and CSs were double-stained as indicated in the figure with antibodies against Ox-DJ-1 (green; **A’’**,**B’’**), (i)sAPPβf (red; **A’**,**B’**), CASP3 (green; **C’’**,**D’’**) and p-Tau (red; **C’**,**D’**) and merged (**A**–**D**). Mean Fluorescence Intensity (MFI) quantification of iAPPβf (**E**), ox-DJ-1 (**F**), p-TAU (**G**), and CASP3 (**H**) reactivity from images obtained by immunofluorescence analysis of PSEN1 WT and I416T CSs. Significant values were determined by *Student’s t-test*; ***p < 0.001. Image magnification 20x.

**Figure 10:**
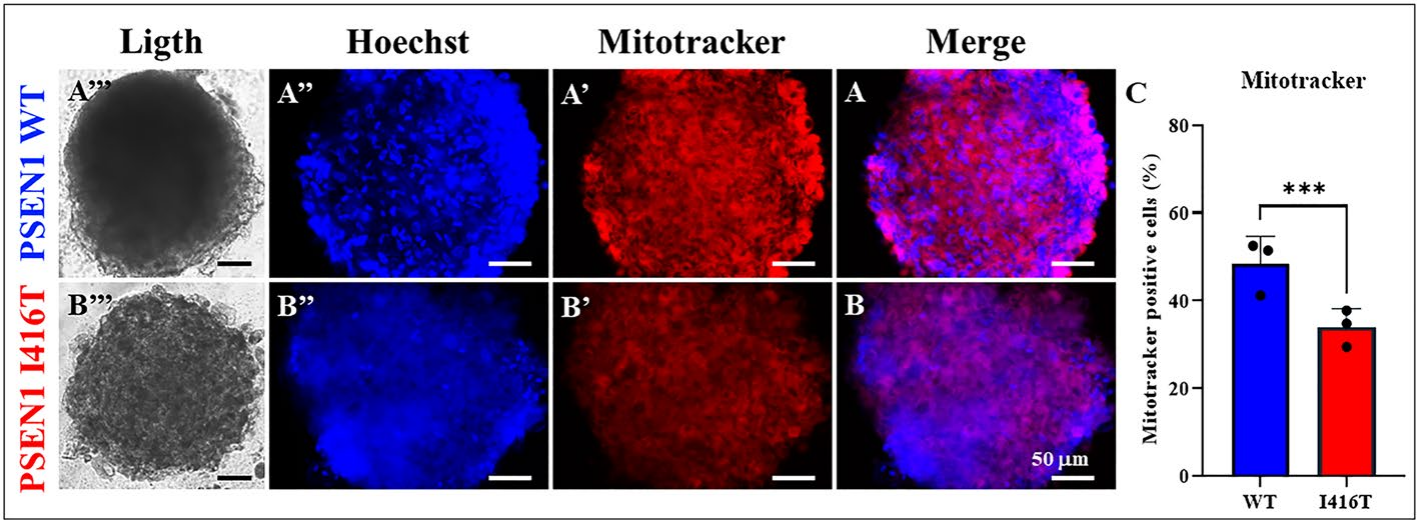
PSEN1 I416T Cerebral spheroids (CSs) show loss of mitochondrial membrane potential (∆Ψm). Both PSEN1 WT and I416T CSs were left in cultured for 11 days. Then, CSs (**A’’’**,**B’’’**) were double-stained as indicated in the figure with Hoechst (blue; **A’’**,**B’’**) and MitoTracker™ Red FM (red: **A’**,**B’**) and merged (**A**, **B**). (**C**) Mean Fluorescence Intensity (MFI) quantification of images obtained by fluorescence image analysis of PSEN1 WT and I416T CSs. Significant values were determined by *Student’s t-test*; ***p < 0.001. Image magnification 20x.

We further investigated whether mutant MenSCs transdifferentiated into CSs and whether CSs can express the pathological markers of FAD. To this purpose, WT and mutant MenSCs were cultured in *Fast-N-Spheres* medium for 11 days^49^. As shown in Fig. 9, both WT and mutant MenSCs transdifferentiated into CSs (Fig. 9A–D), albeit with different cellular profiles. While WT CSs showed none of the amyloid (Fig. 9A'), oxidative stress (Fig. 9A"), p-TAU (Fig. 9C'), or apoptosis marker CASP-3 (Fig. 9C"), the I416T CSs expressed a significant increase in sAPPβf/Aβ aggregates (4.0-f i, Fig. 9B',E), oxidized DJ-1 (8.85-f i, Fig. 9B",F), p-TAU (10.55-f i, Fig. 9D',G), and CASP-3 (516.66-f i, Fig. 9D",H) compared to WT CSs (Fig. 9E–H). Additionally, WT CSs show functional mitochondria membrane integrity (Fig. 10A'), but mutant CSs displayed a significant decrease in the ΔΨ_m_ (1.42-f d, Fig. 10B',C) compared to WT CSs.

### PSEN1 I416T cerebral spheroids (CSs) respond to ACh stimuli

**Figure 11:**
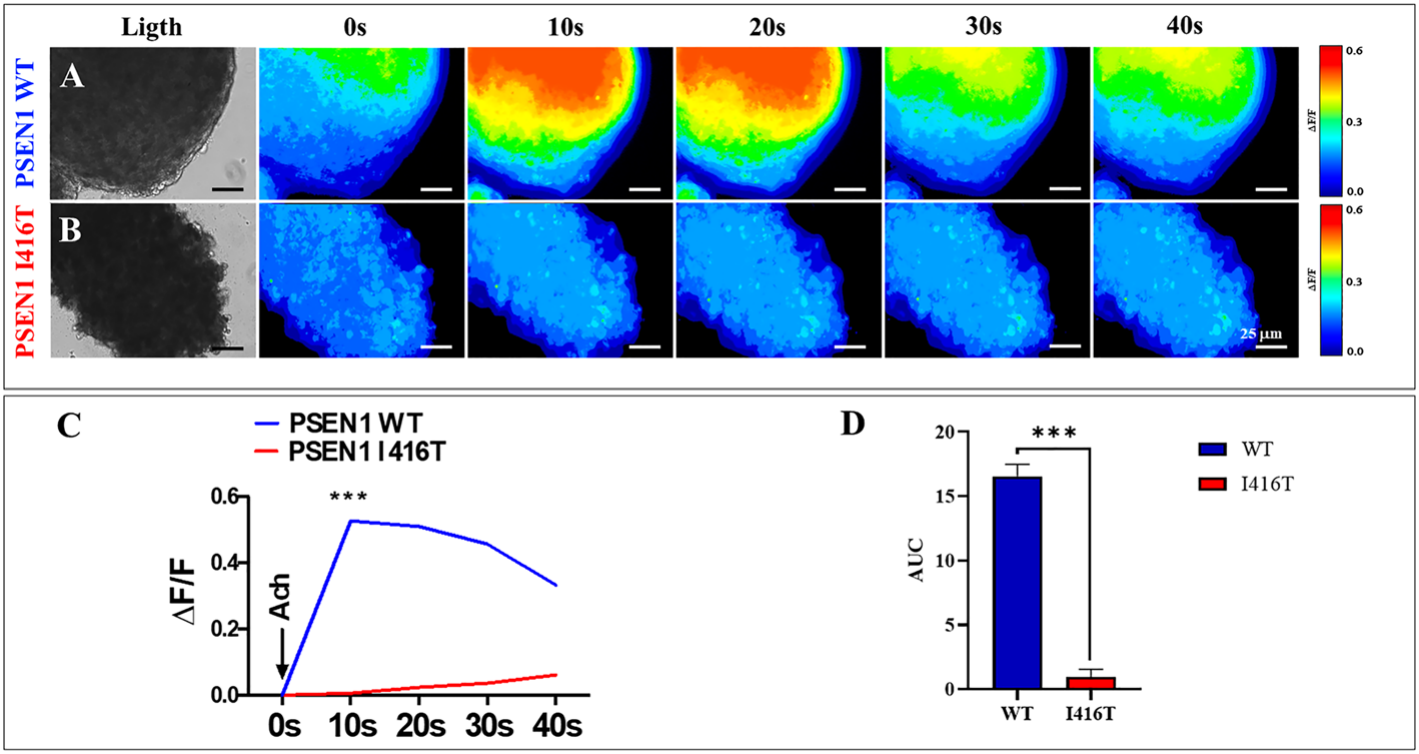
PSEN1 I416T Cerebral spheroids (CSs) show reduced functional response to acetylcholine (ACh). Both PSEN1 WT and I416T CSs were left cultured for 11 days. (**A**, **B**) Time-lapse images (Light, 0, 10, 20, 30 and 40 s) of Ca^2+^ fluorescence in CSs (n = 6 CSs imaged, N = 6 dishes) as a response to ACh treatment. ACh was puffed into the culture at 0 s (arrow). Then, the Ca2 + fluorescence of cells was monitored at indicated times. Color contrast indicates fluorescence intensity: dark blue < light blue < green < yellow < red. (**C**) Normalized mean fluorescence signal (ΔF/ F) over time, indicating temporal cytoplasmic Ca^2+^ elevation in response to ACh treatment in PSEN1 WT and I416T CSs. Significant values were determined by two-way ANOVA with a Tukey post hoc test; ***p < 0.001. Image magnification 20x.

We also tested whether CSs respond to ACh. Fig. 11 shows the response of WT versus mutant CSs when puffed with ACh (1 mM) and recorded for at least 40 s. WT CSs displayed a high transient increase in Ca^2+^ inward (Fig. 11A,C; (ΔF/F) = 0.526 ± 0.02) with a mean duration of 10 s (n = 20 ChLN cells imaged, N = 3 dishes), whereas mutant CSs were almost irresponsive to ACh (Fig. 11B,C, (ΔF/F)= 0.024 ± 0.01) with a similar mean duration of 10 s (n = 20 ChLN cells imaged, N = 3 dishes, Fig. 11D)).

### PSEN1 I416T cerebral spheroids (CSs) secrete high amounts of eAβ_42_

Lastly, we measure the ratio of Aβ_42_ over Aβ_40_ in CSs. As shown in Fig. 7C, mutant CSs produced a significant higher basal level of Aβ_40_ (+2.27-fold) than WT, but PSEN 1 I416T CSs significantly generated more Aβ_42_ (+6.18-f) than WT. The ratio of Aβ_42_ over Aβ_40_ clearly shows that mutant CSs generated more Aβ_42_ (3.28-f) than WT CSs (1.21-f, Fig. 7D).

## Discussion

The development of planar (2D) and tridimensional (3D) in vitro models such as immortalized and iPSCs cell lines, or organ-like models has greatly contributed to the understanding of the cellular and molecular neuropathophysiological features of AD^50–52^**.** However, they are expensive and time-consuming^45,46^. In the present study, we were able to reproduce the neuropathology of AD in MenSCs-derived PSEN1 I416T ChLNs in 2D and 3D (cerebral spheroids) in vitro in 11 days by using *Cholinergic-N-Run*^53^ and *Fast-N-spheres* medium^49^, respectively. We report for the first time that PSEN1 I416T ChLNs displayed the typical intracellular aggregation of protein Aβ (at day 7) and overproduction of eAβ_42_ (at day 11), p-TAU (at day 11), mitochondria damage (at day 11), OS (at day 7), and apoptosis (at day 11). Furthermore, PSEN1 I416T ChLNs but not WT ChLNs were irresponsive to ACh-induced Ca^2+^ inward signal (at day 11). Taken together these findings suggest that intracellular aggregation of Aβ and OS rather than extracellular Aβ are the earliest molecular events involved in neuronal-like cells loss in FAD. In agreement with post-mortem AD aged brain^54,55^, in vitro^27,56^ and in vivo studies^54,57–59^, we found iAPPβf/Aβ aggregates in PSEN1 I416T ChLNs as early as 7 days of MenSCs transdifferentiating. This observation mirrors findings in PSEN 1 E280A^27^. However, the identity of the iAβ species is not yet fully determined. Indeed, investigators have identified either iAβ_42_, iAβ_45_^27^, or iAPPβf^27^, among other Aβ species^60,61^. These findings might be explained by the technique applied for its detection such as immunohistochemistry and immunofluorescence microscopy which uses antibodies against different Aβ conformations (e.g., 6E10, 4G8, anti-Aβ_45_). Interestingly, by using mass spectrometry analysis and immunofluorescence microscopy (6E10), we were able to identify several APP fragments (e.g., APP714, APP733, APP751, APP752, collectively named sAPPβf) but no Aβ_42_ fragment was identified in PSEN 1 E280A ChLNs^27^. Whatever the true nature of Aβ, it accumulates intracellularly^54,62^. Like MSCs-derived PSEN 1 E280A ChLNs, we found intracellular APPβf accumulation in MenSCs-derived PSEN1 I416T ChLNs after 7 days of transdifferentiating (day 0). Interestingly, we also detected Aβ aggregates in PSEN 1 I416T CSs. These observations suggest that intracellular APPβ/Aβ fragments build up because full-length APP processing is altered and consequently some APP fragments remain intracellularly^63,64^. How exactly APPβf/Aβ is generated in PSEN 1 I416T (or E280A and other mutations) is still not yet fully understood. One possible explanation is that the processing of APP is a dynamic biologic process dependent on kinetic enzyme degradation, and cellular location of both β-and γ-secretases. In a normal neural cell, APP is sequentially cleaved by β-secretase (BACE1) freeing a soluble proteolytic fragment, recognized as soluble APPβ (sAPPβ), which might be internalized and delivered to endosomes in the amyloidogenic pathway secreted extracellularly. The remaining C-terminal membrane-bound APP fragment, CTFβ or C99 fragment, undergoes additional cleavages by PSEN1/ γ-secretase to generate mainly Aβ_40_ fragments (90%, nonamyloidogenic) and Aβ_42_ (10%, amyloidogenic). The Aβ peptides are then secreted into the extracellular space when the endosome recycles to the cell surface^65^. In a mutant neural cell, APP is normally cleaved by BACE1 thereby generating APPβf, which by a still unknown mechanism, is stuck intracellularly, probably in early endosomes^66^. Since most mutations in PSEN1, including I416T / E280A, reduced γ-secretase enzymatic activity^33^, it preferentially cuts at γ-site becoming into a major product line^67^ overproducing eAβ_42_^68^. Furthermore, because I416T mutation might also be a trans-dominant negative mutation on γ-secretase^32,69^, the APP-derived eAβ_42_ fragment might be a late event in the process of neuronal-like cell dismiss. Following this view, we detected early iAPPβf/iAβ (at day 7), and late eAβ_42_ (at day 11) in I416T 2D and 3D. Indeed, expression of the I416T mutation increased Aβ_42_ levels but decreased Aβ_40_ levels, resulting in overall increase in the Aβ_42_/Aβ_40_ ratio compared to those WT PSEN 1. Interestingly, it has been demonstrated that the PSEN1 L166P and G384A mutations cause re-localization of γ-secretase in MNT-1 cells (highly pigmented human melanoma cells), which significantly promotes the generation of intracellular long Aβ_42_^70^. Therefore, our data suggest that PSEN 1 I416T mutation strongly enhances intraneuronal APPβf/Aβ aggregates in both ChLNs and CSs, thereby triggering a variety of intracellular signaling mechanisms leading to neuronal-like cell death. In agreement with others^71^**,** these data suggest that iAPPβf/Aβ accumulation is the first step of a lethal cascade.

Previously, it has been shown that oxidation of the oxidative sensor protein DJ-1^106^-SOH (*thiolate*) into DJ-1^106^-SO_3_ (*sulfonic acid)* by H_2_O_2_ and production of ROS were simultaneous events to the detection of Aβ aggregates in MSCs-derived PSEN 1 E280A ChLNs^27^. Here, we observed a similar phenomenon in both MenSCs-derived ChLNs I416T and CSs. However, the biochemical source of ROS/ H_2_O_2_ generated by APPβf/Aβ is not yet defined. Mounting evidence suggests that Aβmight interact with mitochondria, inducing disruption of the electron transport chain, thereby leaking electrons to oxygen, increasing ROS production, and H_2_O_2_^72^. Interestingly, ROS (H_2_O_2_) production was high on day 0 than day 4 post-transdifferentiating, whereas loss of mitochondrial membrane potential and pathological markers expression was observed in day 4. These observations suggest that H_2_O_2_ generation is a cellular transient event (short-lived) and highly reactive towards other molecules via oxidative modification of critical redox-sensitive Cys (e.g., DJ-1)^73^. Once it has directly/ or indirectly activated target molecules including transcriptional factors (TFs), mitogen-activated protein kinases (MAPKs) and protein Tyrosine phosphatases^74,75^, it fades away. Taken together these observations comply with the notion that H_2_O_2_ behaves as a second messenger^74,76^. In line with those findings, mitochondria-specific accumulation of amyloid-β/ H_2_O_2_ induces mitochondrial dysfunction leading to apoptotic cell death^77^**.** Effectively, we found that ChLNs I416T and CSs showed a significant loss of ΔΨ_m_, and expressed several markers associated with regulated cell death. Indeed, ChLNs I416T displayed 2 important active pro-apoptotic transcription factors: c-JUN^78^, and P53^79^**.** Interestingly, both c-JUN and P53 induce apoptosis by direct transcriptional activation of the pro-apoptotic BH3-only protein PUMA^80–82^. Once PUMA is up-regulated, it frames mitochondria to mitochondria outer membrane permeabilization (MOMP) allowing the release of pro-apoptogenic protein (e.g., cytochrome c) and activation of executer protein CASPASE-3^83^. As expected, PSEN 1 I416T ChLNs and CSs showed concurrent loss of ΔΨ_m_ and overexpression of CASPASE-3, indicative of cell death by apoptosis. In agreement with others^57,84–86^, our data suggest that APPβf/Aβ induces apoptosis in I416T ChLNs and mutant CSs. Despite overwhelming observations, other investigators have suggested that iAβ induces other modes of neuronal cell death such as oxytosis/ferroptosis^87^, autophagy^88^**,** and necrosis^89^. We, therefore, do not discard the possibility that I416T mutation might induce not only apoptosis but other supplementary cell deaths in ChLNs. However, further studies are necessary to enlighten this issue.

Hyperphosphorylation of protein TAU appears as a critical marker of neurodegeneration in AD^90^. Indeed, p-TAU at Ser^202^/Thr^205^ is known to be increased in postmortem brains of AD^91^**,** and p-TAU levels increases at later stages of AD^92^. We detected p-TAU (Ser^202^/Thr^205^) in both MenSCs-derived I416T ChLNs and CSs after 11 days of transdifferentiation but APPβf/Aβ aggregation at day 7. In agreement with others^93,94^, our data suggest that APPβf/Aβ precedes p-TAU. A similar conclusion was reached with MSCs-induced PSEN 1 E280A ChLNs^27^. However, how exactly APPβf/Aβ triggers hyperphosphorylation of TAU is not yet established. One possibility is that APPβf/Aβ induces phosphorylation of TAU through c-JUN N-terminal kinase (JNK) signaling^95^**.** Since JNK can be phosphorylated and activated both pro-apoptotic protein c-JUN and TAU at (Ser^202^/ Thr^205^), this kinase has become a potential therapeutic target^96^**.**

Calcium fluxes are intimately involved in neuronal functionality^97^**.** Expectedly, neuronal calcium dyshomeostasis has been implicated in AD as disrupted Ca^2+^ could induce synaptic deficits and memory loss^98^**.** We found that both PSEN 1 I416T ChLNs and CSs were almost irresponsive to ACh-induced Ca^2+^ influx compared to WT ChLNs/ CSs. One possible explanation is that eAβ_42_ might impair the interaction ligand-receptor by direct inhibition of alpha 7 nACh receptors^99^. Following this view, we found that PSEN 1 I416T ChLNs and CSs generated high amounts of eAβ_42_. Although, the nature of AChRs in I416T neuronal-like cells are not yet known, the fast response of ChLNs to ACh-induced Ca^2+^ influx in WT neuronal-like cells suggests that WT and mutant ChLNs and CSs expressed pre-synaptic nAChRs^100^. Indeed, eAβ_42_ affects Ca^2+^ neuronal-like cells inwards. Therefore, acetylcholine receptor constitutes a potential therapeutic target for FAD.

## Conclusion

The I416T is the second most frequent mutation in PSEN 1 in Colombia^34^**.** Interestingly, the clinical phenotype of this mutation resembles the clinical features of mutation PSEN 1 E280A, the most common mutation in the “Paisa” region^8^. Although neuropathological examination of postmortem brains and biochemical studies are still limited for the I416T, we demonstrate in vitro that PSEN 1 I416T MenSCs-derived ChLNs and CSs mirror the typical pathological features of sporadic AD and PSEN 1 E280A FAD^27^ such as intracellular aggregation of APPβf, secretion of eAβ_42_, and phosphorylation of protein tau (Table 1). Although E280A and I416T mutations are structurally apart in the protein PSEN 1, our observations suggest that I416T also disables the catalytic action of PSEN 1/γ-secretase. However, whether the change of an isoline (hydrophobic residue) for a threonine amino acid (uncharged residue) at codon 416 disrupts the 3D structure of PSEN 1 as E280A does (acidic to hydrophobic residue)^30^ needs further investigation. Whatever the structural change, I416T triggers iAPPβf aggregation, eAβ_42_, and p-TAU in ChLNs/ CSs irrespective of ethnic origin (e.g., African vs European (E280A)). Moreover, this mutation causes OS and cell death by apoptosis in ChLN and CSs reflected by a significant increase in oxidation of sensor protein DJ-1, activation of pro-apoptotic protein c-JUN, and P53, expression of PUMA, and activation of executer protein CASP3. The present findings naturally mirrored for the first time the neuropathological features of FAD PSEN 1 I416T. Given that I416T resemble the pathological effects of E280A, and this last mutation decreased both Aβ_40_ and Aβ_42_ but the ratio Aβ_42_/Aβ_40_ favor Aβ_42_^33^, we anticipate that other PSEN-1 related mutation identified in Colombia (e.g., P117A, I143T, H163A)^34^ might result in similar phenotypes as I416T/ E280A mutations wherein there is an overproducing of iAPPβf in ChLNs or CSs. It would be interesting to test whether other mutations which produce Aβ_42_ less than (e.g., D333G, T354I, N405S, A409T) or equal to WT PSEN 1 (e.g., I439V), or mutations with extremely high production of Aβ_42_ (G384A)^33^, are also alike to I416T phenotype. Given the origin of MenSCs, the present observations are limited to female gender. However, for confirmatory purposes, MSCs derived from male tissue (e.g., dental pulp, adipocyte tissue) should be included in future experimental settings. Further investigation is guaranteed on these issues. However, we consistently found iAPPβf in both as I416T/ E280A ChLNs as early as 7 days of transdifferentiating. These observations might contribute to the understanding of the recurring failures of clinical trials of anti-eAβ^101^, and support the view that FAD is triggered by the accumulation of other intracellular APP metabolites, rather than eAβ42^102^. Therefore, other alternative treatment approaches should be pursued for FAD.

## Methods

### Isolation and characterization of menstrual stromal cells (MenSCs) derived from human menstrual blood (MB)

The present study was approved by University of Antioquia, Medellín, Colombia. The menstrual blood samples were collected from a healthy (Tissue Bank Code, TBC # 69308) and asymptomatic FAD (TBC #45000) female aged between 18 and 25 years. Donors provided a signed informed consent approved by the Ethics Committee of the Sede de Investigación Universitaria -SIU-, University of Antioquia, Medellín, Colombia (Act # 19–10-846). All experiments and/or experimental protocol/s were performed in accordance with relevant guidelines and regulations approved by University of Antioquia, Medellín, Colombia. Menstrual blood (MenB) was collected by cup collection (10–15 mL) during the first 3 days of menses. Briefly, menstrual blood samples were delivered into the laboratory and mixed with an equal volume of phosphate-buffered saline (PBS) containing 1 mM ethylenediamine tetra-acetic acid (EDTA), with 100 U/ml penicillin/streptomycin 0.25 mg/ml amphotericin B, and subject to cell lysis or standard Ficoll procedures within 24 h as previously described by^103^. After centrifugation, the cells suspended in a buffy coat (7.7 × 10^6^ ± 3 × 10^6^ cells, n = 3) were transferred into a new tube, washed in PBS twice, and suspended in growth medium (low-glucose DMEM medium supplemented with 10% FBS (Gibco, USA), 100 U/ml penicillin/streptomycin 0.25 mg/ml amphotericin B) and seeded into 25 cm^2^ plastic cell culture flasks at 37 °C with 5% humidified CO_2_. The medium was replaced every 3 days leaving behind the adherent cells that were growing as fibroblastic cells in clusters. When the cells reached 80–90% confluence (passage 0, P0), the cells were detached by 0.25% trypsin/1 mM EDTA and sub-cultured to new flasks by the ratio of 1:3. The isolated MenSCs were evaluated for their differentiation capacity into an osteoblast, chondrocyte, and adipocyte lineage as well as for the presence of cholinergic-like neuronal markers (e.g., choline acetyltransferase, ChAT; vesicular acetylcholine transporter, VAChT) according to^53,104,105^**.**

### Identification of the PSEN1 I416T mutation in MenSCs

The PSEN1 I416T mutation was detected by PCR using mismatch primers and digestion of the products with VspI (AseI isoschizomer) according to^35^. Digested products were separated on a 3% agarose gel. According to different mobility electrophoretic patterns, samples were classified as wild-type (WT, 340 and 220 bp bands) or mutant PSEN1 I416T (560, 340 and 220 bands) when compared to PSEN1 I416T carrier (positive DNA (control) case NeuroBank (NB) code #2495). The TBC #45,000 (female) sample was identified for PSEN1 I416T mutation, whereas TBC #69,308 (female) sample was identified for WT PSEN1. For comparative purposes, we included E280A PSEN1 DNA sample (TBC# 271) as an internal control.

### Cell differentiation

#### Osteogenic differentiation

Osteogenic differentiation was performed according to^104^ with minor modifications. Briefly, WT and mutant MenSCs at passages 4–7 were plated at a density of 10,000 cells/ cm^2^ in 12-well plates in a regular culture medium. After 72 h, the culture medium was replaced by osteogenic differentiation medium containing high-glucose DMEM (Sigma), 10% FBS, 1 µM dexamethasone (Alfa Aesar, cat # A17590), 250 µM sodium ascorbate (Sigma, cat # A4034), and 10 mM β-glycerophosphate (Alfa Aesar, cat # L03425). The medium was changed every 3–4 days. Control cells were kept in regular culture medium (RCm). After 20 days of induction, cells were fixed in 4% FA and incubated with the Mouse Anti-human osteocalcin monoclonal antibody (1:500; R&D, cat# MAB1419) followed by incubation with anti-mouse DyLight™ 594 secondary antibody (1:500) and 1 μM Hoechst 33,342 (Life Technologies).

#### Adipogenic differentiation

Adipogenic differentiation was performed according to^104^ with minor modifications. Briefly, WT and mutant MenSCs at passages 4–7 were plated at a density 20,000 cells/cm^2^ in a 12-well plate in a regular culture medium. At 90–100% confluence, the culture medium was replaced by adipogenic induction medium, including high-glucose DMEM, 10% FBS, 0.5 mM 3-isobutyl-1-methylxanthine (Sigma, cat # I5879), 100 µM indomethacin (Sigma, cat # I7378), 0.1 µM dexamethasone and 10 µg/ mL insulin. Control cells were kept in regular culture medium. After 20 days of induction, cells were fixed in 4% FA and incubated with the goat anti-mouse fatty acid-binding protein 4 (FABP4) antigen affinity-purified polyclonal antibody (1:500; R&D, cat# AF3150), followed by incubation with anti-goat DyLight™ 594 secondary antibody (1:500) and 1 μM Hoechst 33,342 (Life Technologies).

#### Chondrogenic differentiation

Chondrogenic differentiation was performed according to^105^ with minor modifications. Briefly, 5 × 10^4^ WT and mutant MenSCs were left aggregated in microwell plates and then provided with chondrogenic medium containing high-glucose DMEM, 10% FBS, 10 μg/ L TGF-β3, 0.1 μmol/ L dexamethasone, 50 μmol/ L vitamin C, and 6.25 mg/L insulin. The medium was changed every 3–4 days. Control cells were kept in a regular culture medium. After 20 days of induction, cells were fixed in 4% FA and incubated with the goat anti-human aggrecan antigen affinity-purified polyclonal antibody (1:500; R&D, cat# 967,800) followed by incubation with anti-goat DyLight™ 594 secondary antibody (1:500) and 1 μM Hoechst 33,342 (Life Technologies).

#### Cholinergic-Like Neurons (ChLNs) differentiation

The MenSCs were seeded at 1.6–2 × 10^4^ cells/ cm^2^ in 25 cm^2^ culture flasks for 24 h in regular culture medium. Then, the medium was removed and cells were incubated either in minimal culture medium (MCm) or cholinergic differentiation medium (*Cholinergic-N-Run* medium containing DMEM/F-12 media 1:1 Nutrient Mixture Gibco (cat# 10,565,018, 10 ng/ mL), basic fibroblast growth factor (bFGF) recombinant human protein (Gibco cat# 13,256,029), 50 µg/ mL sodium heparine (Sigma-Aldrich cat# H3393), 0.5 µM all-trans retinoic acid, 50 ng/ mL sonic hedgehog peptide (SHH, Sigma cat# SRP3156) and 1% FBS) at 37 °C for 0, and 4 days according to^53^.

#### Cerebral Spheroids (CSs) formation

MenSCs were seeded at a density of 1.5 × 10^4^ cells/cm^2^ in a multi-well plate (Greinner-Bio-one, cat# 662102) using *Fast-N-spheres* medium (DMEN F-12 GIBCO®, cat#11,330–032; supplemented with 2% B27® GIBCO® (cat #17504-044), 20 ng/ml basic fibroblast growth factor (bFGF, R&D Systems, Inc., MN), 20 ng/ml epidermal growth factor (EGF, Sigma cat#E9644), 1 µg/ml heparin sodium salt®, and 100 U/ml penicillin/streptomycin for 11 days according to^49^**.**

#### Immunofluorescence analysis

For the analysis of neural-, Alzheimer disease-, oxidative stress- and cell death-related markers, the cells treated under different conditions were fixed with cold ethanol ( − 20 °C) for 20 min followed by Triton X-100 (0.1%) permeabilization and 10% bovine serum albumin (BSA) blockage. Cells were incubated overnight with primary neural antibodies against choline-acetyltransferase (ChAT, 1:500, cat# AB144 P, Millipore) and vesicular acetylcholine transporter (VAChT, 1:500, cat# SAB4200559, Sigma); primary antibodies against APP751 and/or protein amyloid β1–42 (1:500; clone 6E10 cat# 803014, Biolegend), total TAU (1: 500; t-Tau; cat# T6402, Sigma), and phospho-TAU (p-Tau, 1:500, Ser202/Thr205, cat#MN1020 (AT8), Thermo Fisher Scientific); and primary antibodies against oxidized DJ-1(1:500; ox(Cys106)DJ1; spanning residue C106 of human PARK7/DJ1; oxidized to produce cysteine sulfonic (SO_3_) acid; cat # MABN1773, Millipore). To assess cell death, we used primary antibodies against p53-upregulated modulator of apoptosis (1:500; PUMA, cat# ab-9643, Abcam), p53 (1:500; cat# MA5-12-453, Millipore), phospho-c-Jun (1:250; c-Jun (S63/73) cat#sc-16312, Santa Cruz), and caspase-3 (1:250; cat # AB3623, Millipore). After exhaustive rinsing, we incubated the cells with secondary fluorescent antibodies (DyLight 488 and 594 horse anti-rabbit, -goat and -mouse, cat DI 1094, DI 3088, and DI 2488, respectively) at 1:500. The nuclei were stained with 1 μM Hoechst 33,342 (Life Technologies), and images were acquired on a Floyd Cells Imaging Station microscope. The fluorescence intensity was calculated according to^106^ using the Image J program (https://imagej.net/)^107^.

### Analysis of cells

#### Evaluation of intracellular reactive oxygen species (ROS) by fluorescence microscopy

To determine the levels of intracellular ROS, we used 2’,7’-dichlorofluorescein diacetate (5 μM, DCFH2-DA; Invitrogen) according to^108^. ChLNs were left in regular culture medium (RCm) for 0, and 4 days. Then, the cells (5 × 10^3^) were incubated with the DCFH2-DA reagent for 30 min at 37 ˚C in the dark. Cells were then washed, and dichlorofluorescein (DCF) fluorescence intensity was determined by analysis of fluorescence microscopy images^109^. The nuclei were stained with 0.5 μM Hoechst 33,342 (2.5 μM) staining compound. The assessment was repeated three times in independent experiments blind to experimenter.

#### Evaluation of intracellular reactive oxygen species (ROS) by flow cytometry

ROS was determined with 2’,7’-dichlorofluorescein diacetate (1 μM, DCFH_2_-DA) according to^108^. ChLNs were left in RCm for 0, and 4 days. Then, the cells (1 × 10^5^) were incubated with DCFH_2_-DA reagent for 30 min at 37 ˚C in the dark. Cells were then washed, and DCF fluorescence was determined using an LSRFortessa (BD Biosciences). The assessment was repeated 3 times in independent experiments. Quantitative data and figures were obtained using FlowJo7.6.2 Data Analysis Software. The assessment was repeated three times in independent experiments blind to experimenter and flow cytometer analyst^110^.

#### Analysis of mitochondrial membrane potential (ΔΨ_m_) by fluorescence microscopy

The ChLNs and CSs were left in RCm for 0, 4 or 11 days, respectively. Then, the cells (5 × 10^3^) were incubated with the passively diffusing and active mitochondria accumulating dye deep red MitoTracker compound (20 nM, final concentration) for 20 min at RT in the dark (Invitrogen, cat # M22426) according to^111^. Cells were then washed twice with PBS. MitoTracker fluorescence intensity was determined by analysis of fluorescence microscopy images. The nuclei were stained with 0.5 μM Hoechst 33,342 (2.5 μM) staining compound. The assessment was repeated three times in independent experiments blind to experimenter and flow cytometer analyst.

#### Analysis of mitochondrial membrane potential (ΔΨ_m_) by flow cytometry

ChLNs were left in RCm for 0, and 4 days. Then, the cells (1 × 10^5^) were incubated for 30 min at RT in the dark with MitoTracker (20 nM, final concentration) according to^111^. The cells were analyzed using an LSRFortessa (BD Biosciences). The experiment was performed three times in independent experiments, and 10,000 events were acquired for analysis. Quantitative data and figures were obtained using FlowJo 7.6.2 Data Analysis Software. The assessment was repeated three times in independent experiments blind to experimenter and flow cytometer analyst.

#### Measurement of Aβ1–42 peptide in culture medium

The level of Aβ1–42 peptide was measured according to a previous report^112^ with minor modifications. Briefly, WT and PSEN1 I416T ChLNs or CSs were left in RCm for 4 or 11 days, respectively. Then, 100 μl of conditioned medium was collected, and the levels of secreted Aβ1–42 peptides were determined by a solid-phase sandwich ELISA (Invitrogen, Cat# KHB3544) following the manufacturer’s instructions. The assessment was repeated three times in independent experiments blind to experimenter.

#### Intracellular calcium imaging

The cytoplasmic Ca^2+^ concentration ([Ca^2+^]_i_) was measured according to ref.^113^**.** Briefly, ChLNs cultured in *Ch–N-Rm* for 0, and 4 days, and CSs cultured in *Fast-N-spheres medium* for 11 days were transferred to a bath solution (NBS; in mM: 137 NaCl, 5 KCl 2.5 CaCl_2_, 1 MgCl_2_, 10 HEPES, pH 7.3, and 22 glucose) containing a Ca^2+^ sensitive indicator (2 µM Fluo3-AM, an acetoxymethyl ester form of the fluorescent dye Fluo-3; Thermo Fisher Scientific Cat F1242) for 30 min at RT and then washed five times. The intracellular Ca^2+^ transients were evoked by acetylcholine (ACh, 1 mM final). Before starting the recordings, several ‘‘regions of interest” (ROIs) were defined in the visual field of the camera. One of the ROIs was cell-free, and the fluorescence intensity measured here was considered as ‘‘background fluorescence” (F_bg_). In the ‘‘kinetic view” mode, the program calculated and displayed the average fluorescence intensities of the ROIs in arbitrary units (AUs), as the function of time. In the ‘‘image view” mode, the time dependence of the spatial distribution of the fluorescence emission was shown, the fluorescence intensities (hence the Ca^2+^ levels) were represented either by pseudocolours. To calculate the changes of the average Ca^2+^-related fluorescence intensities in the ‘‘kinetic view” mode, first the F_bg_ value was determined from the cell-free ROI, then the resting fluorescence intensities (F_rest_) of the cell-containing ROIs were obtained as the average of the points recorded during a period of 10 s prior to adding ACh. The peaks of the fluorescence transients were found by calculating the average of three consecutive points and identifying those points that gave the highest average value (Fmax). The amplitudes of the Ca^2+^ related fluorescence transients were expressed relative to the resting fluorescence (ΔF/ F) and were calculated by the formula ΔF/F = (F_max _− F_rest_)/(F_rest _− F_bg_). The fluorescence intensity was calculated as described by^106^ using the Image J program (https://imagej.net/)^107^.

#### Photomicrography and image analysis

Light microscopy photographs and fluorescence microscopy photographs were taken and analyzed exactly as previously reported by^27^ using a Zeiss Axiostart 50 Fluorescence Microscope equipped with a Zeiss AxioCam Cm1 and (Zeiss Whlk-Contact-Linsfluoreen, Gmb Schconkirchen, Germany) and Floyd Cells Imaging Station microscope. Mean fluorescence intensity (MFI) was obtained by normalizing total fluorescence to the number of nuclei.

#### Data analysis

This experimental design was performed exactly as previously reported by^49^ based on the statistics considerations described in by^114^. Given that the experimental unit (i.e., the well) data fulfill the independence of observations, the dependent variable is normally distributed in each treatment group (Shapiro–Wilk test), and variances are homogeneous (Levene’s test), the statistical significance was determined by Student’s t-test, one-way, or two-way ANOVA followed by Bonferroni’s or Tukey’s post hoc comparison calculated with GraphPad Prism 5.0 software. Differences between groups were only deemed significant when a *p*-value of < 0.05 (*), < 0.001 (**) and < 0.001 (***). All data are illustrated as the mean ± S.D.

### Ethical approval

Menstrual specimen donors provided a signed informed consent approved by the ethics committee of the Sede de Investigación Universitaria (SIU), University of Antioquia, Medellín, Colombia (Act 2020-10854).

### Consent to participate

Informed consent was obtained from all individual participants included in the study.

### Supplementary Information

Supplementary Information.

## Data Availability

All datasets generated for this study are included in the manuscript.
