# Supplementary material for: Cholinergic-like neurons and cerebral spheroids bearing the PSEN1 p.Ile416Thr variant mirror Alzheimer's disease neuropathology
Source: Sci Rep. 2023 Aug 8;13:12833. doi: 10.1038/s41598-023-39630-4 (PMC10409854; doi:10.1038/s41598-023-39630-4)
Supplement: Supplementary file 1 — Supplementary Information. [file 41598_2023_39630_MOESM1_ESM.pdf]

Nicolas Gomez-Sequeda, Miguel Mendivil-Perez, Marlene Jimenez-Del-Rio, Francisco Lopera, and Carlos Velez-Pardo. Cholinergic-like neurons and cerebral spheroids bearing the PSEN1 p.Ile416Thr variant mirror Alzheimer's disease neuropathology. Sci Reports

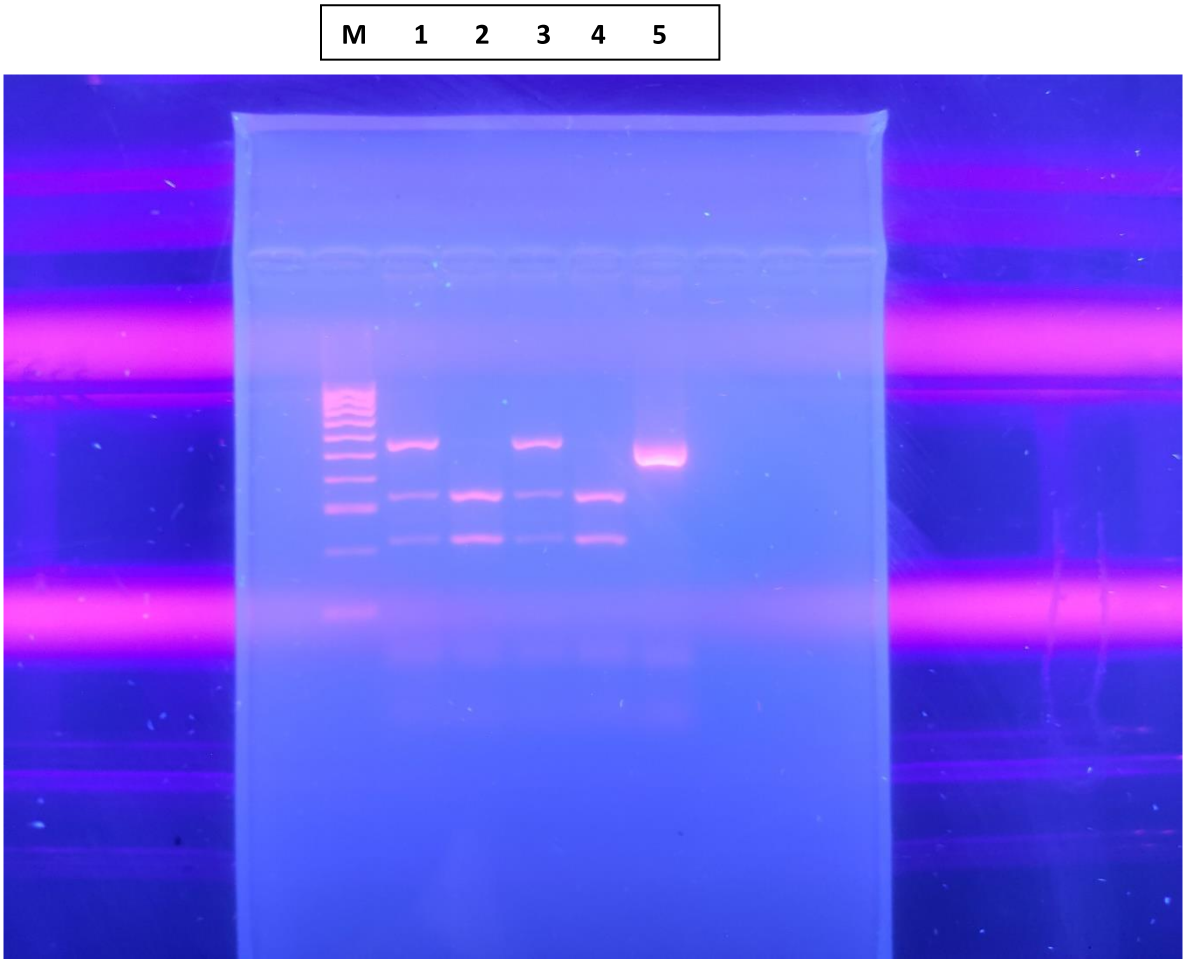

Supplementary Figure 1. Agarose gel electrophoresis for the I416T mutation isolated from samples of mutation carriers. PCR products from blood samples were subjected to restriction endonuclease digestion analysis using AseI enzyme, resolved on 2% agarose gel electrophoresis, and visualized with red gel under ultraviolet illumination. The size of the amplicon was 560 bp. The 340 and 220 bp fragments correspond to the wild-type phenotype, and the 560, 340, and 220 fragments are for the mutant heterozygous phenotype. (M) Molecular weight marker; track (1) positive control with the I416T mutation (Code 2495); track (2) Wild phenotype negative control (Code 69308); track (3) mutation carrier (Code 45000); track (4) mutation carrier E280A (Code 271); track (5) control of digestion (Code 4500, without the AseI enzyme); track (6) PCR control (without template).
